# Labial Repositioning Using Print Manufactured Polymethylmethacrylate- (PMMA-) Based Cement for Gummy Smile

**DOI:** 10.1155/2021/7607522

**Published:** 2021-12-21

**Authors:** Patricia Freitas de Andrade, Jonathan Meza-Mauricio, Ricardo Kern, Marcelo Faveri

**Affiliations:** ^1^Independent Researcher, Brazil; ^2^Department of Periodontology, Dental Research Division, Guarulhos University, Guarulhos, SP, Brazil

## Abstract

Treating patients with excessive gingival display (EGD) to provide them with a pleasant smile is a challenge to periodontists. A gummy smile can be due to excessive vertical bone growth, dentoalveolar extrusion, short upper lip, upper lip hyperactivity, or altered passive eruption (APE). In addition, many patients have a lack of lip support due to marked depression of the anterior process of the maxilla. In these cases, lip repositioning using polymethylmethacrylate (PMMA) cement could be performed. This article describes a case of EGD with subnasal depression. In the clinical examination, the presence of a marked subnasal depression was found, in which the upper lip lodged during a spontaneous smile. In addition to this, gingival exposure extending from the maxillary molar on one side of the mouth to the one on the opposite side was also found during the spontaneous smile. Therefore, the periodontal surgical intervention proposed consisted of performing a procedure to fill the subnasal depression with PMMA cement. This article describes a digital approach to plan the use of PMMA cement in lip repositioning in a patient with gummy smile and subnasal depression. The patient reported no postoperative complications. Six months after the surgery, the patient revealed a more harmonious smile than before, with reduction in the gingival exposure and new adequate support for and repositioning of the upper lip.

## 1. Introduction

Attaining a “perfect smile” has become a major goal for many people of this era. With the mouth being the focus of communication, the smile plays a significant role in expression and appearance [1]. Different developmental or acquired conditions can affect these structures and cause patient dissatisfaction.

One of the most frequent conditions related to an unesthetic smile is the presence of excessive gingival display (EGD), otherwise known as a gummy smile. This condition is defined as a high smile line showing more than 1.5 to 2 mm of the gingiva on smiling [2]. EGD is generally considered unattractive, and while there is a discrepancy in the range defined as excessive, 3 mm has been agreed on across different populations [3].

EGD is a multifactorial condition that may result from a single discrepancy or interplay of several discrepancies, and etiologic factors may be broadly defined as being dentoalveolar and nondentoalveolar [2]. Dentoalveolar discrepancies include those that affect dentition in the form of short clinical crowns, gingival overgrowth, altered passive eruption, and extrusion [4]. These discrepancies are less challenging to treat as they involve orthodontic treatment and restorative and periodontal plastic surgery procedures [5], whereas nondentoalveolar discrepancies involve vertical maxillary excess and a hyperactive, incompetent, or short lip [2]. For the challenging cases of these patients, a multidisciplinary approach such as orthognathic surgery, botulin toxin injections, lip repositioning surgery, or combination of therapies can sometimes be beneficial to enhance the balance and harmony among the three components of the smile (the teeth, lip framework, and gingival scaffold [5, 6]). Furthermore, many patients have a lack of lip support due to marked depression of the anterior process of the maxilla [7]. In these cases, esthetic clinical crown lengthening may be combined with lip repositioning with the use of polymethylmethacrylate (PMMA) cement [8].

The PMMA is a cross-chain polymer material that has a good degree of compatibility with human tissues, and it has been used in several medical and dentistry surgeries [8–11]. Therefore, this article is aimed at describing a digital approach to plan the use of PMMA cement for the purpose of lip repositioning in a patient with gummy smile and subnasal depression.

## 2. Case Description and Case Management

Written informed consent was obtained from the patient for publication of this case report and any accompanying images. This article followed the CARE statement for description of clinical cases [12].

**Figure 1:**
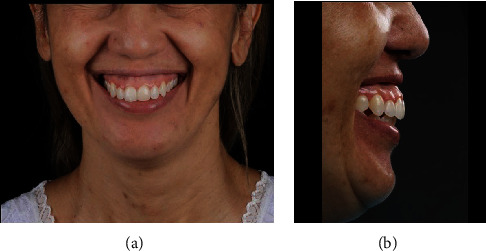
(a, b) Preoperative view of patient's smile.

The patient, a 47-year-old woman, visited a private dental practice in Sao Pablo (Brazil), complaining of “a large exposure of the gums when smiling.” After an extraoral, labial, periodontal, and radiographic evaluation, the presence of a marked subnasal depression, in which the upper lip lodged during a spontaneous smile, was found (Figures 1(a) and 1(b)). In addition to this, gingival exposure extending from the maxillary molar on one side of the mouth to the one on the opposite side was also found during the spontaneous smile (Figure 1(a)). The periodontal surgical intervention proposed consisted of performing a procedure to fill the subnasal depression with PMMA cement and clinical crown lengthening of tooth 12 in order to improve the proportion between the teeth. The patient signed a written term of free and informed consent before undergoing the surgical treatment.

**Figure 2:**
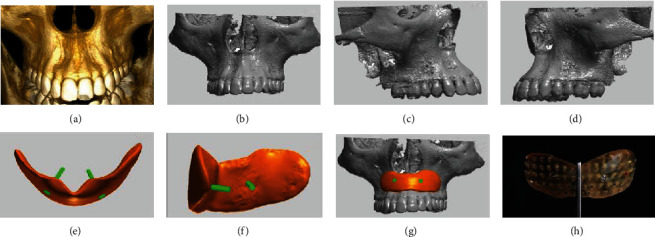
(a) Cone-beam computed tomography scan was performed to evaluate the subnasal depression and to plan the PMMA block. (b–g) Planning of the PMMA block in a 3D planning software according to the defect geometry. (h) Printed PMMA block.

A cone-beam computed tomography (CBCT) scan was performed to evaluate alveolar bone anatomy (subnasal depression) and plan the position, size, and shape of the PMMA block (Figure 2(a)). These data were submitted to the 3D planning software (Materialise Mimics) in the Digital Imaging and Communications in Medicine (DICOM) format to generate a digital model of the bone defect situation and enable virtual planning of the PMMA block in order to achieve an optimal fit to the defect geometry. It was established that the thickness (volume) of the PMMA block must not extend beyond the bone envelope. The digital planning also allowed planning of the long term the screw positions to prevent the screws from interfering with roots or vital structures (Figures 2(b)–2(e)). The digital planning was forwarded to us for evaluation and was consistent with our expectations (Figures 2(e)–2(g)). After confirmation of the design and approval for production, the PMMA (Formslab) block was printed according to the digital data provided by using a 3D printer (Objet V260) (Figure 2(h)). Finally, this block was sterilized in ethylene oxide before surgery.

**Figure 3:**
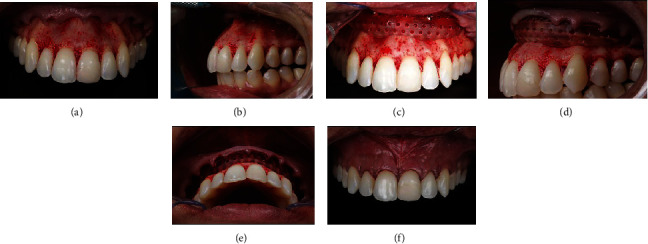
(a, b) View of subnasal depression. (c–e) The PMMA block was fixed with two titanium-based bone graft fixation screws. (f) The gingival flap was repositioned with vertical mattress sutures.

The periodontal surgery was performed under local anesthesia (Lidocaine Nova DFL, Rio de Janeiro, RJ, Brazil). The gingivectomy was performed with an internal bevel incision using a # 15C blade only on tooth 12. Subsequently, intrasulcular incisions were made, and a Molt instrument was used to raise a full thickness flap and to expose the entire frontal region of the maxilla. This allowed access to the subnasal depression and the anterior nasal spine (Figures 3(a) and 3(b)). Osteotomy and osteoplasty were performed using spherical burs at high speed and copious saline irrigation. Excess bone tissue was removed for a distance of 3 mm between the bony crest and the new gingival margin, to safeguard the supracrestal tissue attachment.

**Figure 4:**
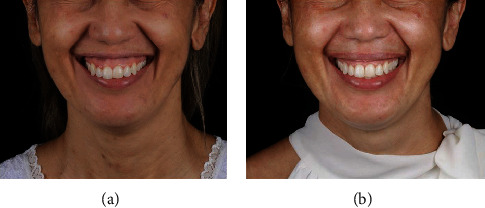
(a, b) Six months later: postoperative view of patient's smile.

After crown lengthening, the PMMA block was fixed with two titanium-based bone graft fixation screws, measuring 1.5 mm × 10 mm (Neodent, Group Straumann, Curitiba, Brazil). One screw was fixed between teeth 11 and 12 and the other between teeth 22 and 23 (Figures 3(c)–3(e)). Finally, the gingival flap was repositioned with vertical mattress sutures using 6-0 nylon (Figure 3(f)). Antibiotics, anti-inflammatory medication, and analgesics were prescribed, and the patient was instructed with regard to postoperative care. At the time interval of 14 days after the surgery, the sutures were removed. The patient reported no postoperative complications. Six months after the surgery, the patient revealed a more harmonious smile than before, with reduction in gingival exposure and new support for and repositioning of the upper lip (Figures 4(a) and 4(b)).

## 3. Discussion

This article describes the treatment of gummy smile in a patient with subnasal depression. The periodontal surgical intervention proposed consisted of performing a procedure to fill the subnasal depression with PMMA cement. This article describes a digital approach to plan the use of PMMA cement in lip repositioning.

Many therapeutic alternatives are available for correction of the “gummy smile.” For each etiology, the best treatment is chosen. In cases of passive eruption, depending on the type, gingivectomy and gingivoplasty procedures, with or without bone resection, are indicated [13]. When the change is due to excessive vertical maxillary growth, several techniques can be used, ranging from orthodontic intrusion through to orthognathic surgery [5]. When the etiology is upper lip hyperactivity, myectomy can be performed, which is resection of the muscles responsible for the mobility of the lip or an application of Botox [6]. Injecting overactive muscles with measured quantities of botulinum toxin results in a reduction of muscle activity, relaxing the lip muscles and decreasing the upward pull on the lip [1]. The improvement achieved is almost immediate but only lasts for a period of 3–6 months, before slowly fading. This relatively short duration is the major disadvantage of the technique, thus requiring constant reapplication [1].

The new technique of filling the subnasal depression with PMMA cement, which has the function of supporting and reducing movement of the lip, completely improves the esthetics of the smile [8].

The PMMA, a cross-chain polymer material that has a good degree of compatibility with human tissues, has been used in several medical and dentistry surgeries [8–11]. The properties of PMMA, such as inertness, low cost, rigidity, easy preparation, and biocompatibility, make PMMA cements suitable for use in different health-related situations, areas of the body, and procedures, such as in cranioplasty, in which it has been used since the second world war [14]. PMMA has been the most widely used material for correcting bony defects of the skull and face, as well as for maxillofacial prostheses and fixation of mandibular fractures [10, 11].

Among the complications associated with PMMA, the long-term rate of infection ranged from 6.25% and 13.9% of the patients [15, 16]. However, Kumar et al. found no complications in any of their 15 patients [14]. In the clinical case presented herein, the PMMA cement was used to fill the subnasal maxillary skeletal depression, thereby repositioning and allowing a new support of the upper lip, contributing to harmonization of the smile that was initially achieved by using clinical crown lengthening. No intraoperative and/or postoperative complications were detected.

The first article in which PMMA was used for subnasal depression in cases of vertical maxillary excess was published by Torres et al. They manipulated and adapted the bone cement directly on the bone surface [7]. In our study, we proposed and described a new approach using 3D print for the use of PMMA cement to achieve lip repositioning. Prefabrication of the PMMA, as was done in this case report, had several advantages. These included complete polymerization, resulting in gain of operative time, and ensuring the achievement of improved physical properties, such as compressive, impact, and shear strength. Moreover, the technology involved was simple to use and easily accessible. To our knowledge, this is the first clinical study that has described the use of printed PMMA in the treatment of gummy smile in a patient with subnasal depression. The results achieved using this approach appeared to be encouraging and could contribute to harmonization of the smile. However, the main limitation of this study was that we only evaluated one clinical case. Therefore, future clinical trials are necessary in order to reach more significant and conclusive results regarding the outcome and long-term stability of this technique.

## 4. Conclusion

The use of PMMA cement could be considered a successful approach for the treatment of EGD in cases with subnasal depression.
